# Traumatic Fracture of the Thoracic Spine With Severe Posterolateral Dislocation: A Case Report

**DOI:** 10.7759/cureus.23830

**Published:** 2022-04-04

**Authors:** Konstantinos Zygogiannis, Konstantinos Manolakos, Anastasios Kalampokis, Georgios C Thivaios, Savvas Moschos

**Affiliations:** 1 Trauma and Orthopaedics, Laiko General Hospital of Athens, Athens, GRC; 2 6th Orthopedic Department, KAT Hospital, Athens, GRC; 3 Spine Department, KAT Hospital, Athens, GRC

**Keywords:** major trauma, fracture-dislocation, spondyloptosis, thoracic, spinal cord injury

## Abstract

Thoracic spine fracture-dislocation injuries are always associated with high-energy trauma and result in severe neurological symptoms. Surgical reconstruction and stabilization are essential for the early mobilization and rehabilitation of patients with this type of injury. Here, we present a unique case of a 57-year-old Greek male who sustained a posterolateral T3-T4 fracture-dislocation, with a complete spinal cord injury (SCI), after falling from a height. The patient was treated with surgical reduction and internal fixation with screws and rods, with satisfactory subsequent realignment.

## Introduction

Traumatic spondyloptosis, or grade V spondylolisthesis, is one of the most severe and rare forms of spinal cord injury (SCI). It is defined as more than 100% traumatic subluxation of adjacent vertebral bodies in the coronal or sagittal plane [1]. It has been classified as fracture-dislocation in the Denis classification of spinal fractures and is considered a very unstable injury, as all spinal columns are disrupted, resulting in complete loss of alignment [2].

Injuries of the thoracic spine represent 21% of all spinal cord injuries (SCI). Traumatic spondyloptosis to the thoracic spine is an extremely rare injury, because of the rigid configuration of the region [3,4]. A great force is needed to overcome its inherent stability, which is mainly attributable to the sternum, the ribs, the anterior longitudinal ligament, the posterior longitudinal ligament, the posterior ligamentous complex, and the sagittal orientation of facet joints preventing axial and horizontal translation [5].

The spinal cord of the thoracic spine is a well-protected structure but very vulnerable to traumatic or vascular insults. Unstable spinal fractures are often associated with complete SCI, particularly in the thoracic region, where there is minimal space around the nerve structures due to the narrow canal and the fragile vascularity of the region [4,6]. Immediate and, frequently, permanent loss of neurological function occurs after any significant dislocation [3]. In 80% of cases, spondyloptosis is associated with transection of the spinal cord and complete neurological deficit [5].

Typically, spondyloptosis is the result of a high-energy injury, usually a motor vehicle accident or a critical fall, and many patients will also suffer from other associated injuries, such as hemothorax or pneumothorax [4]. The pattern and type of fracture-dislocation may be quite variable. Traumatic dislocation of the thoracic vertebra usually occurs in the sagittal plane, with the upper vertebra sliding anteriorly upon the lower vertebra [3]. Lateral and posterior spondyloptosis have rarely been reported. Early surgical stabilization of fracture-dislocation facilitates timely mobilization and rehabilitation. The present study aims to report a case of posterolateral fracture-dislocation of the high thoracic spine causing complete SCI treated with open reduction and internal fixation with screws and rods.

## Case presentation

**Figure 1:**
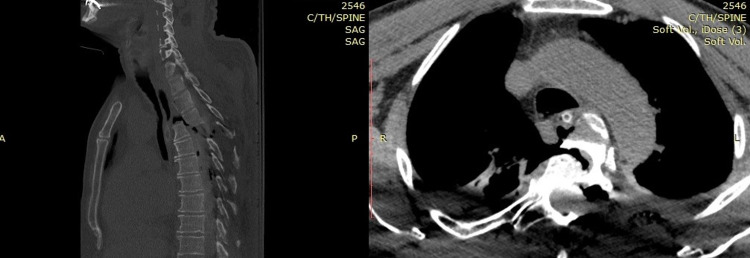
Computed tomography of the thoracic spine Sagittal CT reconstruction of the thoracic spine demonstrating posterior dislocation of T3-T4.

**Figure 2:**
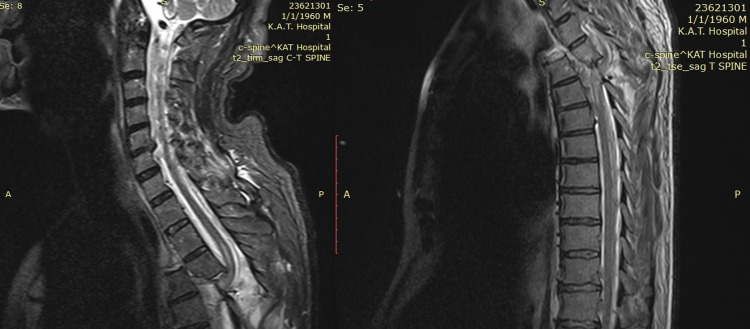
Thoracic MRI of the spine Sagittal T2-weighted MRI (right) demonstrating severe canal stenosis and cord compression at the level of dislocation, and STIR sequence (left) revealing disruption of all posterior ligaments. Intramedullary hyperintensity spanning T3-T4 present in both images likely reflects cord contusion.

A 57-year-old male was admitted to the emergency department after a reported fall from height (20 feet), hemodynamically unstable. Initial neurological examination showed a Glasgow Coma Scale (GCS) score of 15, complete paraplegia, with a grade A American Spinal Injury Association (ASIA) score, and a complete sensory loss below T3. A full-body computed tomography (CT) scan demonstrated a severe posterolateral T3-T4 fracture-dislocation (Figure 1), with multiple costal fractures and bilateral hemothoraces, treated with two chest drains. Magnetic resonance imaging (MRI) showed disruption of all ligaments (anterior longitudinal ligament, posterior longitudinal ligament, ligamentum flavum, interspinous ligaments, and supraspinous ligaments), critical canal stenosis, and cord compression at T3-T4, with associated cord contusion (Figure 2).

**Figure 3:**
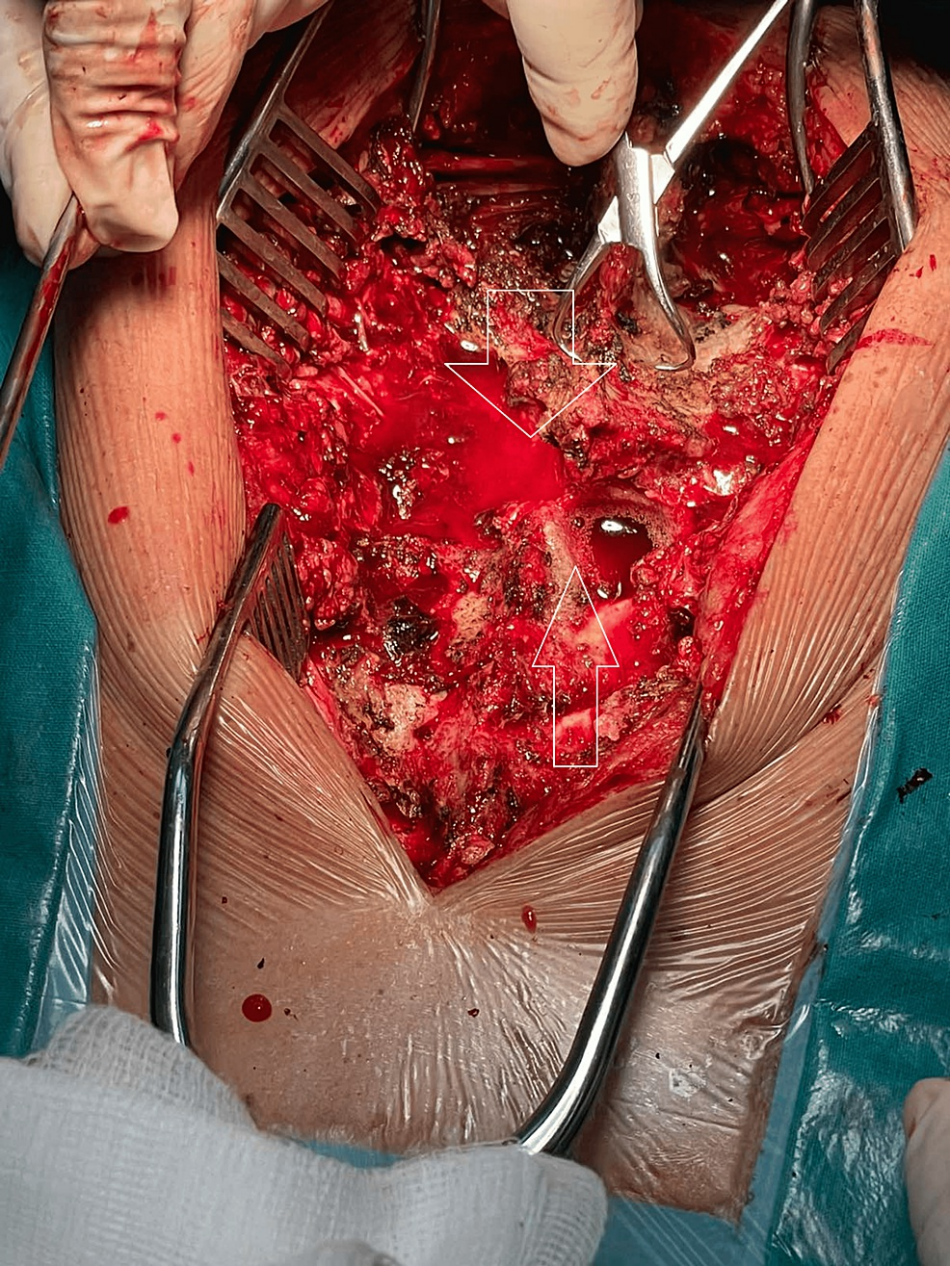
Pre-reduction aspect Pre-reduction position demonstrating significant step-off between T3 and T4 (lower white arrow) and cerebrospinal fluid leakage (upper white arrow).

After 24 hours, as soon as the patient became stable, he was taken to the operating theater. Under general anesthesia and fluoroscopic guidance, the patient was stabilized in a prone position, and a midline posterior incision was made centered on the level of the palpable dislocation. Intraoperatively, after the stripping of the muscles, a significant step-off between T3 and T4 was observed, along with loss of the continuity of the spinal cord with spinal fluid leakage (Figure 3, Video 1).

**Video 1:** Spinal cord disruption Significant spinal cord disruption with spinal fluid leakage.

**Figure 4:**
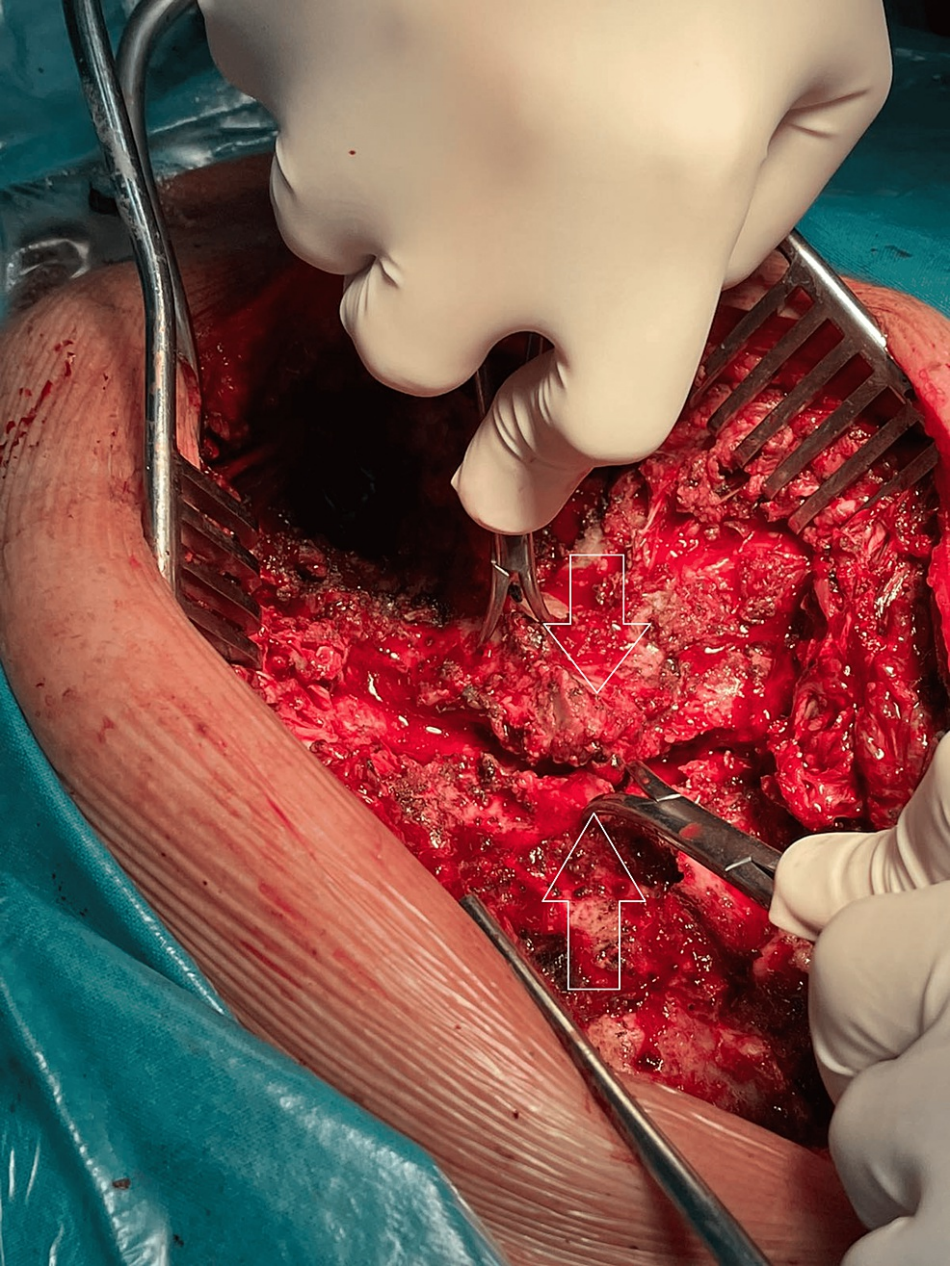
Freehand reduction Attempt to understand if sufficient alignment can be achieved using the freehand technique (white arrows).

**Figure 5:**
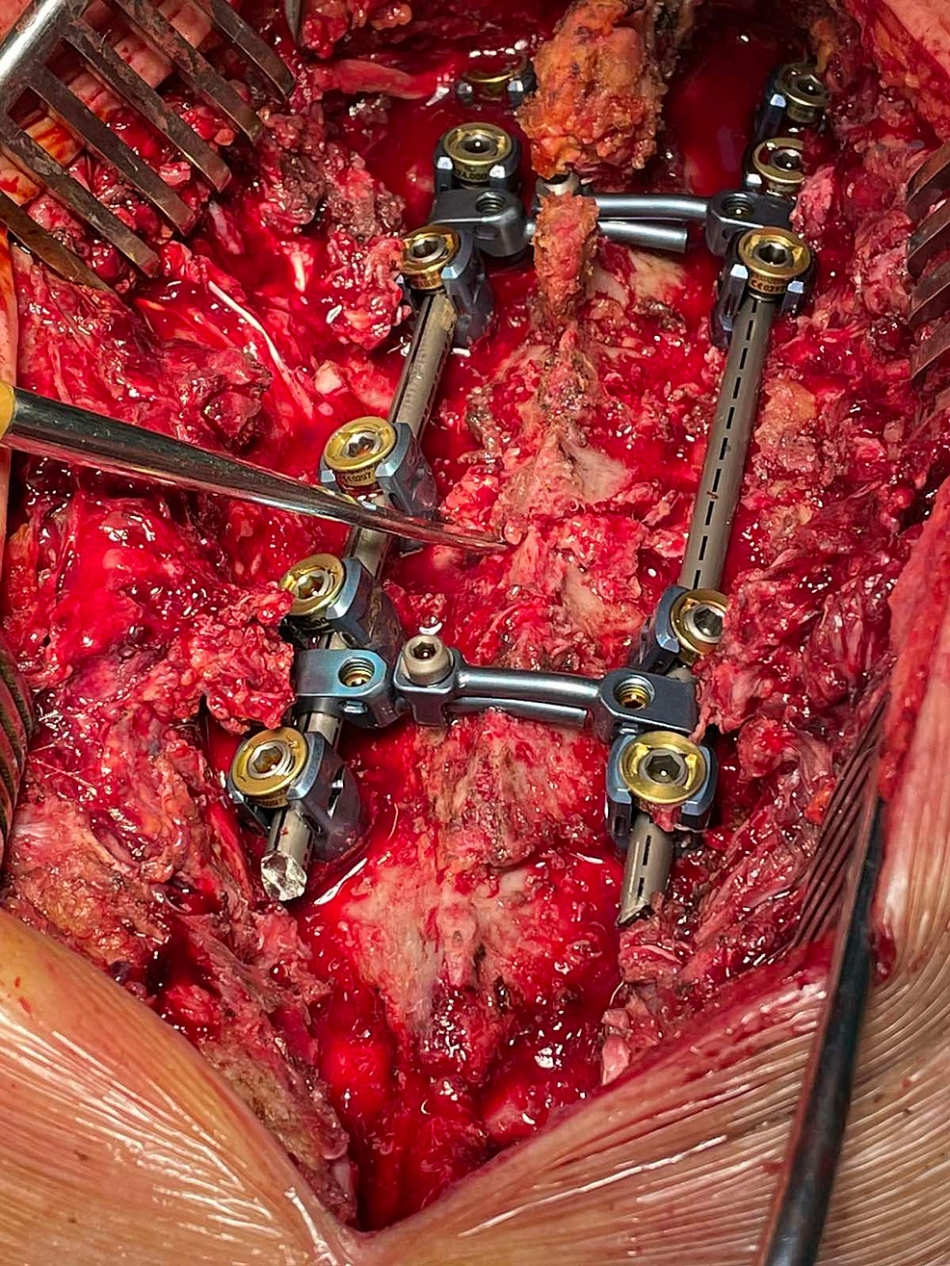
Final construct position with complete reduction of spondyloptosis Sufficient alignment of thoracic fracture-dislocation was achieved due to high instability.

After laminectomy, pedicle screws (Globus Medical, Pennsylvania, USA), 5.5 mm in diameter and 30-45 mm in length, were placed bilaterally from T1 to T7. “Freehand” reduction was attempted and achieved using two Backhaus Towel Forceps (Figure 4, Video 2). Therefore, two rods have been placed and locked, except for T3 and T6, which have been used for compression (Figure 5).

**Figure 6:**
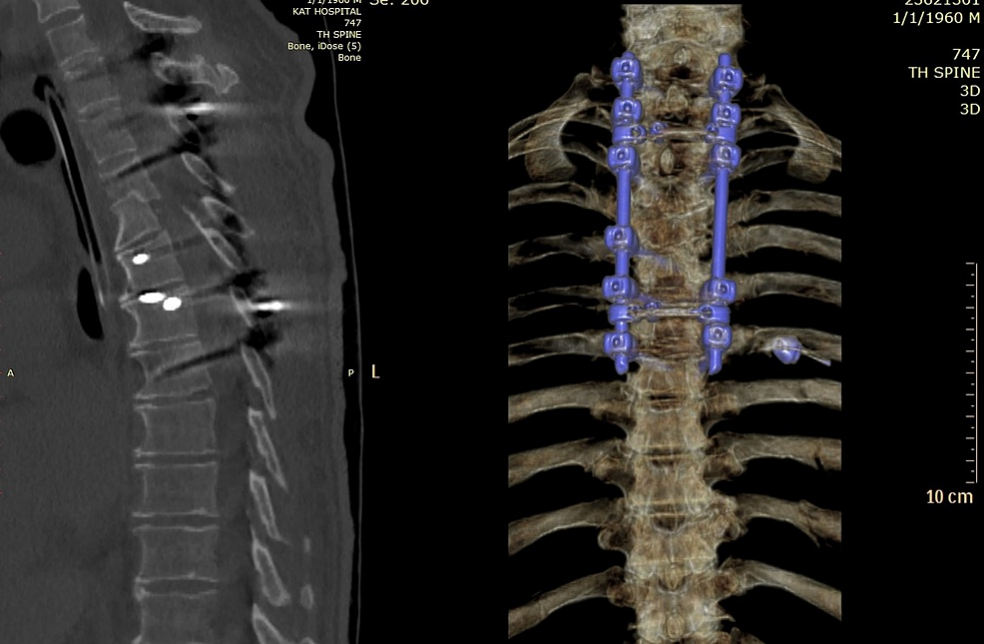
Postoperative CT scan Sufficient alignment of thoracic spine 10 days postoperatively.

Postoperative CT confirmed full reduction of the fracture-dislocation and satisfactory placement of instrumentation (Figure 6). The patient remained paraplegic and ultimately passed away one year after the SCI after a 10-month hospitalization (six months in the intensive care unit).

**Video 2:** Reduction demonstration using Backhaus Towel Forceps

## Discussion

Traumatic thoracic spondyloptosis, defined as more than 100% listhesis of one vertebral body with respect to the adjacent vertebrae either in the coronal or sagittal plane, is one of the most severe types of SCI, associated with more than 80% rate of paraplegia [5]. Most frequently, thoracic spine spondyloptosis occurs anteriorly in the sagittal plane where the superior vertebral body is usually found anterior to the inferior vertebral body. Posterior and lateral spondyloptosis have been rarely reported in the literature [4,7-11]. Hereby, we have described the case of a paraplegic patient with a posterolateral T3-T4 fracture-dislocation treated with internal fixation with screws and rods, without succeeding in restoring the neurological deficit of the patient.

The study by Mishra et al. is the largest case series in the literature, including eight cases of traumatic thoracic spondyloptosis (50% sagittal and 50% coronal). All cases were ASIA grade A at the time of admission and were treated with pedicle screw fixation [3]. The second-largest case series included three patients with thoracic spondyloptosis [12]. Chandrashekhara et al. reported an ASIA grade A patient with T11-T12 fracture-dislocation, managed with pedicle screws and rod fixation, without any neurological improvement [13]. In 2007, Sekhon et al. reported one case of traumatic thoracic spondyloptosis with complete SCI who underwent spinal reduction and posterior stabilization [11]. In four case reports, ASIA grade A patients with thoracic spondyloptosis had no neurological improvement after surgical treatment [7,10,14,15]. Gitelman et al. presented a case of a young male involved in a motor vehicle accident, neurologically intact, with a T6-T7 spondyloptosis. He was treated with bed rest for three months, followed by delayed in situ fusion, with preservation of neurological function [4]. Paulo et al. reported a case of a 17-year-old male with a T11-T12 spondyloptosis treated with posterior decompression, T12 corpectomy, and pedicle screw fixation, resulting in partial improvement of sensory function [16]. A recent study by Li et al. reported a case of a 37-year old male with a posterior T3-T4 dislocation resulting in complete paraplegia. The patient was treated with open reduction with the use of horizontally oriented temporary rods facilitating controlled, sequential sagittal distraction and unlocking, reversal of anteroposterior shear, and restoration of alignment [17].

Curiously, in rare cases, thoracic spondyloptosis may not cause neurological deficit [10,11,18,19]. Neurological status may be preserved by involving free-floating vertebral arches, which spares the spinal cord [4]. In such circumstances, bilateral pedicular shear, designated as saving fractures of the vertebral arch, allows the alignment of the posterior column and ligaments, preserving spinal cord integrity and intact neurological status despite gross displacement of the adjacent vertebral body [9]. Some other studies have reported cases of neurological improvement after spondyloptosis reduction [4,5,12]. However, in all of these cases, the initial neurological status was ASIA grade C or better at the time of presentation. In most cases, the severity of the injury is high enough to establish full paraplegia [3,10-15].

The main goals in the treatment of spondyloptosis are reduction, alignment, and stabilization [7]. In the case of paraplegic patients, the goal of the surgery is early mobilization and rehabilitation [7]. Access to the upper thoracic spine during thoracotomy is difficult due to the anterior presence of the sternum and the posterior presence of the scapula. Moreover, important organs such as the heart and great vessels may be in danger. The surgical treatment of reducible spondyloptosis has usually included open posterior approaches and distraction between segmental pedicle screws in the sagittal plane [3,7], producing sufficient reduction force to allow restoration of alignment. The application of reducers to screw heads along with intradiscal distraction with spreaders has also been described [11]. The fixation of a minimum of three vertebrae below and above the dislocated vertebral bodies is advised [7]. The use of external maneuvers such as manual abdominal pressure or pelvic traction has been effective in some cases [3]. In the case of irreducible spondyloptosis, corpectomy, via either the transpedicular [3,7] or anterior approach, has also been applied, especially in the case of failure of the initial reduction attempt. Despite these treatment approaches, full reduction could not be achieved in about 25% of patients due to the remoteness of the potential proximal and distal fixation points for traction and difficulties accessing the thoracic spine from anterolateral approaches [3,4]. Li et al. have recently reported the parallel placement of multiple horizontal rods for facilitating reduction [17]. We believe that, in such cases, full reduction can be achieved by transpedicular screws and rod compression maneuvers through a single posterior approach.

## Conclusions

In conclusion, thoracic posterolateral spondyloptosis is a rare but important cause of complete SCI. Reduction of severe thoracic spinal fracture-dislocations, traditionally through distraction between pedicle screws, may be proved difficult even in the most experienced hands. Timing and surgical skills are two of the most important assets in order to obtain an optimal result. Depending on the severity of the dislocation, it may prove challenging for open surgical reduction even with or without osteotomy. Available data strongly support that early intervention, especially in patients with thoracic fractures and high injury severity scores, may be most beneficial. With good reduction and proper stabilization, even paraplegic patients may be able to work independently and continue social interactions in a wheelchair.
